# One Soul and Several Faces of Evaporative Dry Eye Disease

**DOI:** 10.3390/jcm13051220

**Published:** 2024-02-21

**Authors:** Antonio Di Zazzo, Stefano Barabino, Romina Fasciani, Pasquale Aragona, Giuseppe Giannaccare, Edoardo Villani, Maurizio Rolando

**Affiliations:** 1 Ophthalmology Complex Operative Unit, Foundation Campus Bio-Medico University Hospital, 00128 Rome, Italy; 2 Ophthalmology Unit, Campus Bio-Medico University, 00128 Rome, Italy; 3 Ocular Surface & Dry Eye Center, ASST Fatebenefratelli SACCO, Kilan Univeristy, 20123 Milan, Italy; stefano.barabino@unimi.it; 4 Ophthalmology Unit, “Fondazione Policlinico Universitario A. Gemelli IRCCS”, 00128 Rome, Italy; romina.fasciani@unicatt.it; 5 Ophtalmology Unit, Catholic University of “Sacro Cuore”, 00128 Rome, Italy; 6 Ophthalmology Clinic, Department of Biomedical Sciences, University Hospital of Messina, 98122 Messina, Italy; paragona@unime.it; 7 Eye Clinic, Department of Surgical Sciences, University of Cagliari, 09124 Cagliari, Italy; giuseppe.giannaccare@unicz.it; 8 Eye Clinic, San Giuseppe Hospital, IRCCS Multimedica, University of Milan, 20123 Milan, Italy; edoardo.villani@unimi.it; 9 Ocular Surface and Dry Eye Center, ISPRE Ophthalmics, 16129 Genoa, Italy; maurizio.rolando@gmail.com

**Keywords:** evaporation, dry eye, ocular surface, tears

## Abstract

The ocular surface system interacts with, reacts with, and adapts to the daily continuous insults, trauma, and stimuli caused by direct exposure to the atmosphere and environment. Several tissue and para-inflammatory mechanisms interact to guarantee such an ultimate function, hence maintaining its healthy homeostatic equilibrium. Evaporation seriously affects the homeostasis of the system, thereby becoming a critical trigger in the pathogenesis of the vicious cycle of dry eye disease (DED). Tear film lipid composition, distribution, spreading, and efficiency are crucial factors in controlling water evaporation, and are involved in the onset of the hyperosmolar and inflammatory cascades of DED. The structure of tear film lipids, and subsequently the tear film, have a considerable impact on tears’ properties and main functions, leading to a peculiar clinical picture and specific management.

## 1. Introduction

The ocular surface system (OSS) includes a complex network of ocular tissues of different structural, functional, histological, and embryological origins. The cornea, conjunctiva, lids, and a multiform lacrimal and secretory system, as well as endocrine, nervous, and immune systems, interact in such a system via the mediating function of the tear film. The tear film, as a multilayered fluid tissue, carries out multiple functions. It has metabolic and optic activities, and a clearance function, but is mostly a networking scaffold for several neuromediators, hormones, immune cells, and cytokines, which regulate ocular surface system homeostasis, also defined as para-inflammation. A healthy anatomic tear film structure and subsequent function are critical components to preserve ocular surface system equilibrium, allowing para-inflammatory mechanisms to properly react to daily repetitive insults, stimuli, and trauma to which the OSS is exposed [1,2,3,4,5].

Therefore, a healthy tear film is the most dynamic component of the ocular surface system and varies depending on whether the eye is open or closed [6], basal or reflex tear production [7], and sick versus normal settings, and it is capable of enabling extremely swift adaptive reactions [8,9]. However, three theoretical and virtual layers are detectable: the outer thinner lipid layer, a deeper and thicker aqueous layer, and an inner final mucous layer, which links and also connects the tear to the epithelium [10,11].

Since it is directly exposed to the environment, the OSS is particularly vulnerable to outside stressors, which mostly causes the evaporation of the tear film once its components are altered or damaged, leading to a failure of OSS homeostatic para-inflammatory mechanisms [12,13].

This failure may be related to a broad and progressive spectrum of conditions. This varies from occasional ocular discomfort to a severe immune-mediated dry eye disease [14], which may be related to a rarer aqueous deficient dry eye disease (ADDE) and/or to a more frequent evaporative dry eye disease (EDE). These are often simultaneously present in the same patient in the mid–long-term disease [15,16]. Therefore, the purpose of our review is to highlight how the structure, composition, and architecture of the tear film are crucial for its function, by exploring the several faces and diversified causes of the evaporative alteration of the tear film and its clinical impacts on patients.

## 2. Physiological Evaporation

Evaporation is the process by which molecules from a liquid escape to the surrounding atmosphere by transitioning into a gas or vapor [17,18]. Evaporation from the tear film is a normal feature of the ocular surface/tear film physiological system, and acts as a stimulus for tear aqueous production and basal eyelid blinking [19,20].

Evaporation induces focused cooling on the surface of the eye, leading to a subsequent cooling effect in the underlying tissues such as the cornea and conjunctiva [18,21]. The sensory nerves distributed across the eye surface pick up on this tissue cooling [22]. This localized cooling plays a role in the feedback mechanisms regulating the production of aqueous tears and the occurrence of normal, spontaneous blinking [19]. Alternatively, evaporation might result in a confined elevation of tear film osmolarity. This increased osmolarity is then sensed by the sensory nerves on the ocular surface, serving as a stimulus for the initiation of tear production and blinking [23].

Evaporation is a constant feature of all liquids, but the extent of evaporation is affected by the volatility of the liquid, the temperature of the liquid, the exposed surface area of the liquid, the presence of other substances in the liquid (which will alter the inter-molecular bond ratios), and the characteristics of the atmosphere directly above the liquid surface, i.e., presence/absence of gas, gas type, temperature, humidity, pressure, and presence of air movement [24,25].

## 3. Evaporation Contributing Factors 

The exact mechanism preventing tear evaporation is not fully understood, but it is likely that an interlocking bipolar inner layer acts as a transitional layer between the polar aqueous tear film and the outside non-polar surface. On top, a thin lipid layer covers the amphipathic surface, preventing evaporation and reducing the surface tension [26,27]. The contribution of tear film lipids to reducing the rate of evaporation has been estimated using the difference between the evaporation rates of water and tears measured in vitro. This is consistent with Mathers’ analysis showing that, although the lipid volume has an impact on the rate of evaporation in individuals with dry eye symptoms, it does not entirely account for it [28,29,30,31,32,33,34]. When evaluating the rate of evaporation of tears in vivo, environmental factors such as air flow, humidity, and temperature must be carefully controlled [35,36]. Human tears evaporate at substantially lower rates than the estimated 10 microliters/minute of water at 34 °C in 30% relative humidity [37]. 

It has been demonstrated in a controlled evaporation chamber (22.3 uC ± SD 0.7; relative humidity: 18.5 ± SD 4.5%; air flow 15 L/min) [38] that prolonged environmental stress causes symptoms typical of DED and reduces tear secretion in otherwise healthy mice. Previous studies have shown that patients with dysfunctional tear syndrome have an increased tear film water evaporation rate compared to subjects with normal eyes, regardless of whether they have normal or low Schirmer test scores [12,13].

Tear clearance from the ocular surface is slowed by increased tear water evaporation and reduced tear production. This may lead to a build-up of toxic tears with high osmolarity and a high concentration of pro-inflammatory chemicals in the lower nasal region of the conjunctiva [4,9]. 

Interestingly, a study showed evaporation was increased in patients with dry eye and accounted for the majority of the tear loss in patients with dry eye. Normal tear osmolarity was assessed even with low tear flow if evaporation was kept within the normal range [29].

Prolonged evaporative stress can cause lacrimal glands to secrete less tear fluid, as demonstrated in animal models [39]. The causes of this are unclear because it occurs quickly in mice, but negative feedback to the gland may occur due to nerve injury, a dry lacrimal sac due to slower tear turnover in the lacrimal excretion pathways, or even dry eyes. A dry eye that is already obviously evaporative gradually develops into one that is also aqueous-deficient. Among other factors, the capacity of a lipid film to spread over a fluid greatly depends on the amount of free water [38], and a shortage of water on the ocular surface may hamper the spread and affect the stability of the lipid layer.

### 3.1. Lipid Layer Thickness

The outer non-polar lipid layer mainly consists of wax esters, cholesterol esters, diesters, triglycerides, diglycerides, monoglycerides, free sterols, and free fatty acids. Just 13–170 nm thick, the effectiveness of this layer is crucial for the stability and function of the tear film [40,41,42,43,44].

The lipid layer is principally sourced in the oils in the meibomian glands located in the upper and lower eyelids [45,46,47,48,49], with blinking spreading the lipids over the tear film [50,51]. Lipid layer thickness (LLT) depends on various factors, including meibomian gland function [41], the composition of meibomian gland secretions, the effectiveness of blinking [52], the width of the interpalpebral fissure, and ambient humidity [53]. The lipids are released by the meibomian glands during each blink and prevent tear evaporation. The quantity of lipids secreted by meibomian glands is affected by neuronal, hormonal, and vascular factors, and reduced lipid production due to meibomian gland dysfunction (MGD) may lead to tear film instability. Alteration or deficiency in the quality, quantity, or spreading ability of tear film lipids causes increased tear evaporation, hyperosmolarity, failure of homeostatic para-inflammatory mechanisms, and long-lasting inflammation [54].

The quality and dispersion of the lipids are more important than their quantity. The fact that only some of the glands are active at any one time suggests that each gland has cycles of activity and quiescence during which acinar reserves are restored. According to certain analyses of dry eye scores, a normal amount of meibum requires only a few secreting glands [55]. Non-polar lipids provide a clean optical surface, act as a barrier against external objects and organisms, and are thought to be the primary component controlling tear water evaporation [56]; despite accounting for a minority of the lipids in the tear film, polar lipids (phosphatidyl ethanolamines, sphingomyelin, and phosphatidyl choline [28]) are crucial for the regulation of tear film stability and surface characteristics since a lower phospholipid/neutral lipid ratio lowers the stability of the lipid film [57,58].

Finally, the thickness of the lipid layer indicates the presence of lipids, but does not accurately reflect their effectiveness or their ability to prevent evaporation. A thin tear film lipid layer may be due to a lipid deficiency, but also to a failure of the lipids to form an efficient elastic bilayer [59].

### 3.2. Lipid Wax Esters

Wax esters (WEs) are crucial in protecting against water evaporation [60,61]. The lipid melting point must be at or slightly above the interfacial temperature in order to slow evaporation [56]. Age-related variations in the composition of meibum (and therefore the stability of the tear film) may be partially explained by the relationship between the physical condition of WEs at specific temperatures and their ability to reduce evaporation. It is possible to hypothesize that a decrease in melting temperature reduces the capacity of WEs to deter evaporation as a result of an altered degree of the saturation and branching of carbon chains. The lipid layer in an infant delays evaporation more efficiently than that of an adult, which also explains the reduced blinking rate of children.

WEs generate lipid layers that are unstable in a dynamic environment. They spread extremely slowly, prefer aggregation over uniform spreading, and are incompressible. They must be combined with more effective surfactants, including polar phospholipids, because phospholipids such as phosphatidylcholine and sphingomyelin are necessary to ensure that they are spread and work properly.

On the basis of these observations, WEs have a number of unique features that may explain the variations in their evaporation retarding effects, especially when they are extremely close to their solid—liquid phase transition. When WEs are solid, a sizeable portion of the interface is not lipid-coated, thus allowing them to float on water like an iceberg. Similarly, when they are extremely fluid-like, considerable lipid vibration creates a significant amount of free space inside the lipid membrane, thus enabling water vapor molecules to pass through the WE membrane [62]. The structure of WEs resembles a monolayer when they are almost at their melting temperature, which slows evaporation [63].

Both mechanisms cause the tear film lipid layer to be unstable and break up quickly, leading to increased evaporation, which clinically causes ocular discomfort in DED. [40,64]. Afterwards, a higher evaporation rate may increase tear film osmolarity [65], which is a clinical and molecular marker of OSS inflammation [66].

### 3.3. Blinking

Blinking spreads the tear film lipid layer. A blink cycle consists of a down and an up stroke (each lasting approximately 0.2 s), the blink itself, and an interblink phase of about 3–4 s [67]. On the skin side of the mucocutaneous junction, meibomian oil is supplied to the marginal reservoirs of the upper and lower lids and, during the upstroke of a blink, it is distributed from the lower reservoir onto the aqueous sub-phase of the tear film in order to re-establish the lipid layer [68]. Any slowing in spreading the lipid layer prolongs the period of exposure of the ocular surface in the interblink period [28], thus supporting the importance of the quality, composition, and distribution of lipids in preventing tear film evaporation. Blinking serves as a rapid and involuntary mechanism to shield the eyes from potential hazards in the environment. The blink reflex is a crucial protective response to sudden threats, such as foreign objects, bright lights, or noxious stimuli.

### 3.4. Aqueous–Mucin Layers

The underlying aqueous tear fluid has a significant impact on lipid layer function, and the spread of the lipid layer may correlate with the amount of tear fluid present in the aqueous compartments of the tear film. The volume of the tear film’s aqueous phase is particularly significant because it contains surfactant-active substances (mucins) that are necessary for the lipid layer to spread and function [69,70,71,72].

Such mucins dispersed in the water portion of the tear film allow lipids to spread on top of the aqueous layer and prevent excessive evaporation from tear film. The role of dissolved mucins in allowing the spread of lipids on the tear surface is critical in overall tear film stability [73].

The aqueous/mucin layer contains certain dissolved glycoproteins that are essential for the lipid spreading. The amount and quality of tear lipids are affected by the condition or dysfunction of the lid-associated glands, which are also a potential source of infection and inflammation. Furthermore, the lipid layer in healthy eyes spreads uniformly immediately after a blink, but its dispersion time is noticeably extended in subjects with aqueous-deficient DED. This is supported by the discovery that there is a substantial negative association between lipid layer stabilizing time and Schirmer’s test, thus indicating that the amount of tear fluid on the ocular surface increases as the lipid layer spreads and stabilizes more quickly [74].

Finally, an epithelial dysfunction caused by friction, unfavorable environmental conditions, ocular surface irritation, and/or nerve damage definitively causes an alteration in the intraepithelial mucin layers, which connect all the tear layers as hardware or a scaffold of the tear film. In addition, impaired nerve function and structural alterations can also have an impact on epithelial viability and turnover, irreparably damaging epithelial-transmembrane mucins’ basal substructure, which is critical for tear film architecture [75].

### 3.5. Oxidative Processes

The ocular surface is exposed to varying amounts of oxidative stress (environmental factors, pollutants, and UV light exposure) for a number of hours every day [76]. Photo-oxidation causes non-enzymatic lipid peroxidation in s-lipid bilayer membranes, and it has been shown that oxidative stress alters the physical characteristics and thickness of phospholipid bi-layers [77].

Reactive oxygen species (ROS), including superoxide anion (O_2_^−^), hydrogen peroxide (H_2_O_2_), singlet oxygen, and hydroxyl radicals (HO•), are continuously generated during the course of normal aerobic metabolism. Tear film lipid layer membranes contain anti-oxidant defenses to nullify the excessive ROS produced during exposure, but chronic ROS exposure can overwhelm the anti-oxidants and other oxidant-degrading pathways.

Bilayer membranes undergo lipid peroxidation as a result of photo-oxidation, which also interferes with their structural organization by altering their thermodynamic characteristics and lipid packing.

The chemical structure of phospholipid fatty acids undergoes significant alterations as a result of lipid oxidation. As the oxidized groups are polar and hydrophilic, they are unlikely to be compatible with the inner, hydrophobic bilayer, which is likely to cause changes in their usual fluidity and ordering, transition temperature, lateral organization, polarity, and permeability. If such changes really occur, it is crucial to identify them because they may affect the way in which the lipid layer regulates evaporation from the ocular surface in an oxidizing environment [78,79].

All such membranes show increased water permeability in comparison with the unoxidized bilayer. Transient transmembrane water holes also occur in stable oxidized bilayers, with pore openings and closings taking place in tens of nanoseconds. This is consistent with the findings of theoretical and experimental studies showing that oxidized lipid membranes have increased permeability [80]. It has been demonstrated that increasing water permeability causes post-oxidation membrane destruction, which may explain why tear film water evaporation increases even in the absence of MGD [79].

The polarity of phospholipid bilayers is also crucial because changes in polarity indicate greater membrane permeability. Water penetration is influenced by the composition of phospholipids; for example, the presence of polar groups in oxidized phospholipids may attract more than the usual number of water molecules into the bilayer, and thus increase local polarity [78,79,80,81,82].

It is particularly intriguing to note that a study of subjects with moderate DED and poor tear stability (a tear thinning time [TTT] of 5–10 s) showed that oral anti-oxidant treatment led to a clinically significant improvement in TTT in comparison with no treatment or placebo (tear stability of >10 s is considered normal) [78,82,83].

Oxidation can also change the behavior of phosphatidylcholine monolayers, as has been demonstrated by fluorescence microscopy [78,79]. However, not all of the chains are reoriented as they were terminated oxidized lipids. Short-chain products were incorporated into stable bilayers and, in the stable systems, there was a decrease in the thickness of the membrane containing aldehyde-terminated oxidized lipid products, and an increase in the systems with hydrocarbon short-chain products. This was due to the hydrophobic nature of the oxidation product molecules, which predominantly resided near the center of the bilayer and thus prevented membrane shrinkage. The thickness of the oxidized bilayer therefore depended on the type of short-chain products, which goes beyond the conclusions of earlier studies that omitted short-chain products and solely considered the reduction in membrane thickness upon oxidation. All of the considered membranes were more water-permeable than the non-oxidized bilayer [79].

### 3.6. Temperature Changes

The development of DED may be aided by a rise in temperature [84]. A plausible hypothesis that dry eye symptoms are caused by increased tear evaporation due to high temperatures is supported by a human study that revealed a significant rate of tear evaporation at high room temperature [85]. This suggests that keeping ambient air at an appropriate temperature and humidity level is better for eye health, particularly in the case of DED patients.

According to a recent study, DED symptom questionnaire scores correlate with ambient temperature, with higher scores in the winter [86]. Low ambient temperatures have been associated with meibomian gland constriction or plugging, which reduces the thickness of the lipid layer and jeopardizes the stability of tear film [87]; they may also be related to DED symptoms by activating cold corneal thermoceptor receptors and triggering ocular pain [88].

Electronic reading devices (tablets and personal computers) increase the number of incomplete blinks, which may be related to ocular discomfort by reducing the integrity of the pre-corneal tear layer rather than lowering blink frequency (BF). However, it seems that BF is partially regulated by the cold stimulation of transient receptor potential cation channel sub-family M, member 8 (TRPM8) thermoreceptors as a result of the local hyperosmolarity induced by increased tear evaporation from local dry hyperosmotic spots and consequent local tear film break-up [89].

### 3.7. Epithelial Alteration

**Figure 1:**
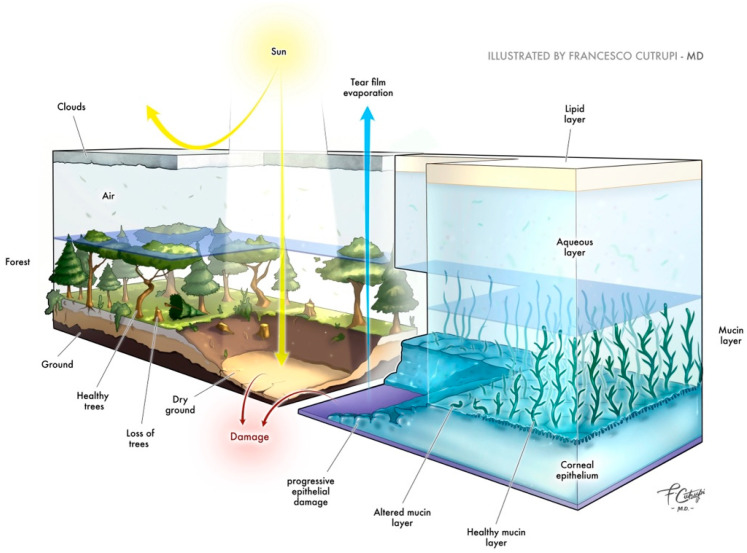
The role of tear evaporation in the pathogenesis of dry eye syndrome. The ocular surface can be regarded as a true ecosystem, where each component is vital for maintaining homeostasis.

A healthy epithelium critically impacts tear stability. An alteration of the cell surface barrier may cause evaporation by directly altering the mucous interaction with tears via the reduction in villi and microvilli. These are critical in increasing the cell–tear interactions. Mostly, however, the reduction in transmembrane mucins causes a collapse in the tear’s architecture, thereby altering the lipid layer [90]. Figure 1 shows how the ocular surface can be regarded as a true ecosystem, where each component is vital for maintaining homeostasis. Alteration in the tear film, particularly in the lipid layer (compared to a layer of clouds), leads to excessive evaporation of the aqueous layer, resulting in the drying out of the surface. This can cause superficial epithelial damage, akin to the harm in a forest lacking protection from intense solar heat.

## 4. Clinical Picture

### 4.1. Low-Tech Diagnosis

An irregular or unstable pre-corneal tear film may cause visual disruption in patients with DED. The tear film usually consists of an aqueous gel and an outer lipid layer that prevents tears from evaporating between blinks [6]. However, in DED patients, a lack of tear production or changes in tear film quality lead to aberrations and scattering, which directly affect the performance of the optical system. Furthermore, the exposure of an irregular corneal epithelial surface caused by tear film break-up significantly increases intra-ocular scattering. Rolando et al. [91] also demonstrated a decrease in spatial contrast sensitivity, and Tutt et al. [3] demonstrated a combination of increased aberrations and scattering during the interblink interval, which lowers the quality of the image projected on the retina.

Therefore, those patients experience a severe reduction in best-corrected visual acuity, which is more detrimental to quality of life than the ocular discomfort they suffer from. Tear film intra-blinking instability worsens the optical quality of the tear film (and then the ocular surface), leading to an intermittent and variable loss of visual quality. Such a condition may be, in severe cases, also described as functional blindness, because affected patients experience a severe limitation in their daily activities, such as driving, sleeping, and working [92].

Subsequently, patients show a compensatory increase in the blinking rate, which allows the tear film to be distributed over the cornea more frequently, thus reducing ocular discomfort and improving picture quality [93,94]. In fact, Parkinson’s-affected patients frequently show decreased blinking rates as well as an increased evaporation rate, which cause ocular discomfort and visual failure [95].

As tear film efficiency has crucial effects on the health of the ocular surface, clinicians and researchers face the challenges of identifying any deficient components of the ocular surface system and enabling the quick and uniform spreading of the lipid layer on the surface of the tear film.

Studies of the causes of dry eyes have revealed a wide range of risk factors, including daily computer use, hormones, the wearing of contact lenses, low humidity settings, cancer treatment, ageing, and a variety of systemic disorders. According to a new analysis, DED is best understood as an overall failure of the mechanism that protects the surface of the eye and allows it to respond to environmental stress [5,96,97].

Several conditions are mainly related or begin with alternative pathogenetic conditions related to increased evaporation. In the case of disease related to sex hormone impairment [73], a critical impairment of the mucin layer is associated with tear instability and subsequent evaporative dry eye disease. Instead, such instability is principally due to a neurogenic chronic inflammation and loss of para-inflammatory mechanisms in long-lasting type I diabetic patients [96], as well as in autoimmune disease. Impaired blinking and subclinical persistent inflammation cause increased evaporation in elder populations, and lead to ocular discomfort and dryness once cataract surgery is performed, mostly in patients older than 75 years.

In younger populations living in polluted and crowded cities, an increased inflammatory response to a pseudo-allergic reaction and conjunctival hyper-reactivity cause tear evaporation by modifying the mucin layer and affect the lipid composition by increasing the oxidative stress [97,98,99].

In chronic autoimmune and allo-inflammatory conditions, the increase in inflammatory stress leads to a subsequent alteration in mucin and lipid structure and composition, followed by an initial aqueous-deficient dry eye. This is also worsened via a vicious cycle in a component of evaporative dry eye.

Measuring the tear water evaporation rate at the time of diagnosis and during the course of the disease makes it possible to monitor the risk of a worsening ocular surface system, suggest an appropriate therapeutic approach, and check the efficacy of the chosen treatment [100,101].

A recent Dry Eye Expert Board publication [102] suggests that three steps are necessary for an accurate diagnosis of DED: (1) administration of a patient symptom questionnaire, (2) slit lamp observation of the ocular surface in order to examine the tear film and any staining suggesting epithelial damage and lid conditions, and (3) testing for tear clearance and corneal sensitivity. The Symptom Assessment in Dry Eye (SANDE) questionnaire may be preferred to quantify the severity, frequency, and any changes in symptoms over time because it is rapid, affordable, reliable, easy to perform, and patient-friendly [103]. It consists of just two questions that patients answer using a 100 mm, horizontal visual analogue scale.

Slit lamp assessment of tear film instability by tear film break up time (T-BUT), tear meniscus height, and vital staining by fluorescein and lissamine green on the ocular surface may give us the critical information about the qualitative and quantitative tear film alteration, as well as the local structural damage and inflammation, respectively.

Tear film osmolarity is a critical marker of evaporation and, indirectly, of tissue inflammation. Although samples of meniscus tears have an osmolarity of 343 mOsm/L [28,104,105], it is likely that the osmolarity of an exposed pre-corneal tear film is significantly higher [106,107]. Liu et al. [108] evaluated the pain brought on by hyperosmolar eye drops, and found that the burning and stinging sensations felt after tear film breakage were similar to those induced by drops of 800–900 mOsm/kg, thus highlighting the significance of evaporation. The reasons for tear film break-up are less clear than those explaining hyperosmolarity, which is clearly due to increased evaporation and/or decreased tear production.

The findings of numerous studies support the hypothesis that evaporation plays a major role in tear film break-up. McCulley and Sciallis demonstrated that meibomian gland expression increases break-up time from 7.0 to 29.4 s in subjects with meibomian keratoconjunctivitis, which is equivalent to the values observed in healthy controls [109]. Craig and Tomlinson have shown that break-up time varies depending on evaporation rate and lipid layer pattern, and proposed a link between break-up time and lipid thickness [28].

### 4.2. High-Tech Diagnosis

Moreover, in order to assess lipid layer performance, morphological tests such as infrared meibography, which allows the observation of the structure of the meibomian gland, should be combined with functional tests such as meibomian gland expression or the dynamic lipid interference pattern (DLIP) test. Tear break-up time, which measures the amount of time between a full blink and the first break in the tear film, is frequently used to assess tear film stability and is a good indicator of the effectiveness of the lipid layer, as a short value is a sign of a poor-quality lipid layer [103,110].

Interferometry studies have examined the thinning of the tear film over a lengthy period of time following blinking in order to throw light on the mechanisms of breakage [40,50,59,111]. For example, if a tear film is originally 3 microns thick (2 s after a blink), it is reduced to 0 thickness in 9 s, for a total of 11 s, which is equivalent to the non-invasive break-up time [112]. The thinning rate is quick enough to explain tear film break-up. Tear film thinning is completely or significantly reduced when wearing air-tight goggles [113] and, of the two potential processes of tear film thinning (tangential flow and evaporation), only evaporation would be abolished or diminished by wearing goggles [114,115,116,117,118]. If evaporation is the primary cause of linear tear film thinning between blinks, it follows that evaporation rather than tangential flow is primarily responsible for tear film break-up time [16,109,119,120]. Tangential flow into the meniscus, which is what causes the thinning and subsequent disintegration of the tear film at the level of the black line close to the meniscus, would be an exception.

Interferometry [121], meibography [122], the DLIP test [123], the meibomian gland expressor test [124], and other technology-related tests [125] have all been created to assist clinicians in identifying lipid layer problems. Non-invasive interferometry evaluates tear film stability, and may also be used to analyze the thickness of the lipid layer, which may be a sign of meibomian gland malfunction (TearScienceVR, LipiViewVR) [120].

The various methods of using interference patterns to measure LLT include the use of a gooseneck slit lamp, a semi-quantitative method [126], biodifferential interference microscopy [127,128], diffuse cold cathode tube illumination, differential interference contrast microscopy, polaroid filters, and monochromatic light [129,130,131]. These measurements have been correlated with tear film evaporation [132], tear film break-up time [28], and the symptoms of ocular discomfort [133].

The development of an easy-to-use, no-touch evaporimeter provides a means of indirectly testing tear film stability and the ability of the lipid layer to resist evaporative stress, the risk of over-stimulation, and the need of the ocular surface to react by means of the adaptive mechanism of para-inflammation in order to adjust and maintain its homeostasis. If it is dysfunctional, para-inflammation may be responsible for the development of clinically chronic inflammation. The instrument could also be used to test treatment efficacy and the ability of the ocular surface system to reduce the inflammatory cascade caused by the over-stimulation associated with excessive evaporation.

## 5. Treatment

Understanding the pathophysiology and persistence of a chronic dry eye condition is crucial for the development of an effective therapeutic strategy, which requires taking into account the way in which evaporation affects the effectiveness of the ocular surface system [134].

Correct and consistent lid hygiene, forced blinking frequently throughout the day to increase the volume and renew the lipids in the lipid layer in front of the tear film, protective glasses, environmental control, and the use of room humidifiers as needed have all been recently recommended. Systemic anti-oxidant treatment has also been suggested as a means of improving lipid quality [135].

Eyelid hygiene, mostly including massages and warm compresses, can be significant in the treatment and prevention of MGD and possibly evaporative DED. However, although effective, it lacks standardization and requires active patient participation in order to ensure treatment adherence. Several medical devices (such as Blephasteam^®^) have been designed to warm and humidify the eyelid and liquify meibum in order to relieve symptoms and prevent relapses. This approach may also be beneficial in the case of evaporative stress [136].

The regular use of lipid-containing eye drops has been proposed as they more closely mimic the aqueous and lipid layers of natural tears. As DED is most frequently associated with the deficient spreading of non-polar lipids, their main purpose is to add polar lipids such as phospholipids to the tear film in order to allow the better spread of WEs. It can be expected that such lipid-based agents will benefit patients with evaporative DED or related ocular surface diseases. The first generation of lipid-containing lubricant eye drops were not widely used because they tended to induce blurred vision, but the newer formulations are more acceptable [137].

The addition of several high-tech scaffold architectures (such as complex structural matrices) or epithelial and tear active metabolites (such as trehalose) to the formulation may significantly improve tear film integrity and may help against oxidative stress and apoptosis [75].

Essential fatty acid supplementation has recently been tested in a randomized, controlled trial comparing daily oral doses of n-3-eicosapentaenoic acid (EPA) [138] or docosahexaenoic acid (DHA) with placebo olive oil. No significant between-group differences were found in terms of the signs and symptoms of DED over 12 months, but the anti-inflammatory properties of the polyphenols contained in olive oil may have been a confounding factor [139,140]. Omega-3 fatty acids may be useful in treating inflammation as a result of the formation of potent anti-inflammatory and pro-resolving lipid mediators [141]. Therefore, this is still a controversial issue.

Most of the physical treatments used today have the aim of increasing meibomian gland activity in the hope that this will increase the volume and improve the performance of the released lipids rather than enhancing the effectiveness of the lipid layer in limiting evaporation.

Intense pulsed light (IPL) has been suggested for the office-based treatment of MGD [142]. Although it may lead to a temporary improvement in objective non-invasive break-up times, a Cochrane systematic review concluded that it is currently unknown whether it is useful in treating the signs or symptoms of evaporative DED, and its meta-analysis underlined that its safety profile in this patient population is also unknown because side effects have not been adequately reported. Clinicians using this method to treat MGD should take into account the limitations of the current data, and fully explain them to patients who may undergo IPL [143].

The use of the automated lid warming and meibomian gland expression of the Lipiflow^®^ instrument is interesting because it has more physiological lid hygiene activity [137]. The only randomized, controlled trial of intraductal meibomian gland probing (MGP) aimed at restoring natural meibum flow did not show that it had any greater benefit than placebo, although the procedure seemed to be safe as no major complications were documented despite frequent self-limited intraoperative bleeding. Any final assessment of the potential impact of this unique therapeutic technique will therefore require further and larger placebo-controlled studies [144].

In addition to interventional procedures, the use of topical and systemic antibiotics (tetracyclines, macrolides, and fluoroquinolones) can help to regulate bacterial flora and decrease the secretion of bacterial lipases in altered lipid secretions and the release of the fatty acids that irritate the glandular ducts.

## 6. Discussion

Ocular surface system failure, and its subsequent dry eye condition, is a multifactorial disease, and clarity concerning its underlying biological mechanism facilitates its diagnosis and early treatment, thus avoiding progression to chronic inflammation and permanent damage to the ocular surface.

Evaporation seriously affects the homeostasis of the ocular surface and plays an important role in the pathogenesis of DED. Tear film lipid composition, distribution, spreading, and efficiency are crucial factors controlling tear film water evaporation, and are involved in starting the hyperosmolar and inflammatory cycle of DED. The function of tear film lipids seems to be affected by lipid oxidation, tear film water volume, and epithelium integrity more than total lipid volume, and their dynamic behavior on the ocular surface has a considerable impact on its anti-evaporative properties.

The concept of failure of the ocular surface system as a critical step in disease development is a novel approach to such patients. The idea of evaporative DED is limited to MGD, which naturally affects the main source of lipids and is associated with a reduction in lipid volume, and is significantly different from the pathogenic process that causes the excessive evaporation of tear film water and DED. However, as we have shown, increased evaporation is not solely due to the quantity of lipids on the ocular surface [13].

As suggested by Korb and Blackie [145], the main cause of the damage and symptoms of the disease should be seen in terms of its risk factors, development, and maintenance to identify the appropriate treatment for the growing number of patients with evaporative and/or secondary aqueous-deficient tear film problems.

The characteristics of such patients and their requirements are still best described using the term syndrome of increased tear evaporation. The existence of continuous evaporative stress is a recurrent stimulus that induces the ocular surface to activate an adaptive response such as para-inflammation; however, the persistent nature of the stimulus easily dysregulates para-inflammation and leads to the subclinical inflammation typical of DED. In order to prevent dry eye from becoming chronic, it is essential to manage evaporative stress and the cues associated with it on the ocular surface.

## 7. Conclusions

Ocular surface system failure, and conversely the subsequent DED, are the latest manifestations of several contributing causes that are strictly correlated. Tear evaporation syndrome is a crucial starting component of such a pathogenesis. An accurate assessment and treatment of the several faces/alterations, which may lead to increased evaporation, are required for better long-term management of this chronic condition.

## Data Availability

Not applicable.

## References

[1] Holly F., Lemp M. (1973). International Ophthalmology Clinics: The Preocular Tear Film and Dry Eye Syndromes.

[2] Stern M.E., Beuerman R.W., Fox R.I., Gao J., Mircheff A.K., Pflugfelder S.C. (1998). The Pathology of Dry Eye: The Interaction between the Ocular Surface and Lacrimal Glands. Cornea.

[3] Tutt R., Bradley A., Begley C., Thibos L.N. (2000). Optical and Visual Impact of Tear Break-up in Human Eyes. Investig. Ophthalmol. Vis. Sci..

[4] Gipson I.K., Argüeso P., Beuerman R., Bonini S., Butovich I., Dana R., Dartt D., Gamache D., Ham B., Jumblatt M. (2007). Research in Dry Eye: Report of the Research Subcommittee of the International Dry Eye WorkShop (2007). Ocul. Surf..

[5] Di Zazzo A., Coassin M., Surico P.L., Bonini S. (2022). Age-Related Ocular Surface Failure: A Narrative Review. Exp. Eye Res..

[6] Sack R.A., Beaton A., Sathe S., Morris C., Willcox M., Bogart B. (2000). Towards a Closed Eye Model of the Pre-Ocular Tear Layer. Prog. Retin. Eye Res..

[7] Fullard R.J., Tucker D. (1994). Tear Protein Composition and the Effects of Stimulus. Adv. Exp. Med. Biol..

[8] Green-Church K.B., Nichols K.K., Kleinholz N.M., Zhang L., Nichols J.J. (2008). Investigation of the Human Tear Film Proteome Using Multiple Proteomic Approaches. Mol. Vis..

[9] Versura P., Nanni P., Bavelloni A., Blalock W.L., Piazzi M., Roda A., Campos E.C. (2010). Tear Proteomics in Evaporative Dry Eye Disease. Eye.

[10] Le Q., Zhou X., Ge L., Wu L., Hong J., Xu J. (2012). Impact of Dry Eye Syndrome on Vision-Related Quality of Life in a Non-Clinic-Based General Population. BMC Ophthalmol..

[11] Miljanović B., Dana R., Sullivan D.A., Schaumberg D.A. (2007). Impact of Dry Eye Syndrome on Vision-Related Quality of Life. Am. J. Ophthalmol..

[12] Rolando M., Refojo M.F., Kenyon K.R. (1983). Increased Tear Evaporation in Eyes with Keratoconjunctivitis Sicca. Arch. Ophthalmol..

[13] Rolando M., Refojo M.F., Kenyon K.R. (1985). Tear Water Evaporation and Eye Surface Diseases. Ophthalmologica.

[14] Barabino S., Aragona P., di Zazzo A., Rolando M., Berchicci L., Bonini S., Calabria G., Cantera E., Gambaro S., Leonardi A. (2021). Updated Definition and Classification of Dry Eye Disease: Renewed Proposals Using the Nominal Group and Delphi Techniques. Eur. J. Ophthalmol..

[15] Bron A.J., Yokoi N., Gaffney E., Tiffany J.M. (2009). Predicted Phenotypes of Dry Eye: Proposed Consequences of Its Natural History. Ocul. Surf..

[16] Isreb M.A., Greiner J.V., Korb D.R., Glonek T., Mody S.S., Finnemore V.M., Reddy C.V. (2003). Correlation of Lipid Layer Thickness Measurements with Fluorescein Tear Film Break-up Time and Schirmer’s Test. Eye.

[17] Craig J.P., Singh I., Tomlinson A., Morgan P.B., Efron N. (2000). The role of tear physiology in ocular surface temperature. Eye.

[18] Tan J.-H., Ng E.Y.K., Rajendra Acharya U., Chee C. (2009). Infrared thermography on ocular surface temperature: A review. Infrared Phys. Technol..

[19] Ding J., Kim Y.H., Sarah M.Y., Graham A.D., Li W., Lin M.C. (2021). Ocular surface cooling rate associated with tear film characteristics and the maximum interblink period. Sci. Rep..

[20] Tomlinson A., Trees G.R., Occhipinti J.R. (1991). Tear production and evaporation in the normal eye. Ophthalmic Physiol. Opt..

[21] Purslow C., Wolffsohn J. (2007). The relation between physical properties of the anterior eye and ocular surface temperature. Optom. Vis. Sci..

[22] Gonzalez-Gonzalez O., Bech F., Gallar J., Merayo-Lloves J., Belmonte C. (2017). Functional properties of sensory nerve terminals of the mouse cornea. Investig. Ophthalmol. Vis. Sci..

[23] Monfared N., Murphy P.J. (2023). Features and influences on the normal tear evaporation rate. Contact Lens Anterior Eye.

[24] Cunningham R., Brann J., Fleming G. (1962). Factors affecting the evaporation of water from droplets in airblast spraying. J. Econ. Entomol..

[25] O’Hare K.D., Spedding P.L. (1992). Evaporation of a binary liquid mixture. Chem. Eng. J..

[26] Mishima S., Maurice D.M. (1961). The Oily Layer of the Tear Film and Evaporation from the Corneal Surface. Exp. Eye Res..

[27] McDonald J.E. (1969). Surface Phenomena of the Tear Film. Am. J. Ophthalmol..

[28] Craig J.P., Tomlinson A. (1997). Importance of the Lipid Layer in Human Tear Film Stability and Evaporation. Optom. Vis. Sci..

[29] Mathers W.D., Daley T.E. (1996). Tear Flow and Evaporation in Patients with and without Dry Eye. Ophthalmology.

[30] Tsubora K., Yamadat M. (1992). Tear Evaporation from the Ocular Surface. Investig. Ophthalmol. Vis. Sci..

[31] Shimazaki J., Sakata M., Tsubota K. (1995). Ocular Surface Changes and Discomfort in Patients with Meibomian Gland Dysfunction. Arch. Ophthalmol..

[32] Goto E., Endo K., Suzuki A., Fujikura Y., Matsumoto Y., Tsubota K. (2003). Tear Evaporation Dynamics in Normal Subjects and Subjects with Obstructive Meibomian Gland Dysfunction. Investig. Ophthalmol. Vis. Sci..

[33] Matsumoto Y., Dogru M., Goto E., Endo K., Tsubota K. (2004). Increased Tear Evaporation in a Patient with Ectrodactyly-Ectodermal Dysplasia-Clefting Syndrome. Jpn. J. Ophthalmol..

[34] Yokoi N., Mossa F., Tiffany J.M., Bron A.J. (1999). Assessment of Meibomian Gland Function in Dry Eye Using Meibometry. Arch. Ophthalmol..

[35] Borchman D., Foulks G.N., Yappert M.C., Mathews J., Leake K., Bell J. (2009). Factors Affecting Evaporation Rates of Tear Film Components Measured in Vitro. Eye Contact Lens.

[36] Rolando M., Refojo M.F. (1983). Tear Evaporimeter for Measuring Water Evaporation Rate from the Tear Film under Controlled Conditions in Humans. Exp. Eye Res..

[37] Hisatake K., Tanaka S., Aizawa Y. (1993). Evaporation Rate of Water in a Vessel. J. Appl. Phys..

[38] Barabino S., Shen L.L., Chen L., Rashid S., Rolando M., Dana M.R. (2005). The Controlled-Environment Chamber: A New Mouse Model of Dry Eye. Investig. Ophthalmol. Vis. Sci..

[39] Barabino S., Rolando M., Chen L., Dana M.R. (2007). Exposure to a Dry Environment Induces Strain-Specific Responses in Mice. Exp. Eye Res..

[40] Nichols J.J., Mitchell G.L., King-Smith P.E. (2005). Thinning Rate of the Precorneal and Prelens Tear Films. Investig. Ophthalmol. Vis. Sci..

[41] Korb D.R., Baron D.F., Herman J.P., Finnemore V.M., Exford J.M., Hermosa J.L., Leahy C.D., Glonek T., Greiner J.V. (1994). Tear Film Lipid Layer Thickness as a Function of Blinking. Cornea.

[42] Korb D.R., Greiner J.V. (1994). Increase in Tear Film Lipid Layer Thickness Following Treatment of Meibomian Gland Dysfunction. Adv. Exp. Med. Biol..

[43] Korb D.R. (2000). Survey of Preferred Tests for Diagnosis of the Tear Film and Dry Eye. Cornea.

[44] McCulley J.P., Shine W.E. (2000). Changing Concepts in the Diagnosis and Management of Blepharitis. Cornea.

[45] Holly F.J., Lemp M.A. (1977). Tear Physiology and Dry Eyes. Surv. Ophthalmol..

[46] Brown S.I., Dervichian D.G. (1969). The Oils of the Meibomian Glands. Physical and Surface Characteristics. Arch. Ophthalmol..

[47] Tiffany J.M. (1979). The Meibomian Lipids of the Rabbit. I. Overall Composition. Exp. Eye Res..

[48] Tiffany J., Marsden R., Holly F. (1986). The Influence of Composition on Physical Properties of Meibomian Gland Secretion. The Preocular Tear Film in Health, Disease, and Contact Lens Wear.

[49] Nicolaides N., Holly F. (1986). Recent Findings on the Chemical Composition of Steer and Human Meibomian Glands. The Preocular Tear Film in Health, Disease, and Contact Lens Wear.

[50] Brown S.I., Dervichian D.G. (1969). Hydrodynamics of Blinking. In Vitro Study of the Interaction of the Superficial Oily Layer and the Tears. Arch. Ophthalmol..

[51] Wolff E. (1946). The Mucocutaneous Junction of the Lidmargin and the Distribution of the Tear Fluid. Trans. Ophthalmol. Soc. UK.

[52] Dilly P.N. (1994). Structure and Function of the Tear Film. Adv. Exp. Med. Biol..

[53] Korb D.R., Greiner J.V., Glonek T., Esbah R., Finnemore V.M., Whalen A.C. (1996). Effect of Periocular Humidity on the Tear Film Lipid Layer. Cornea.

[54] Baudouin C., Messmer E.M., Aragona P., Geerling G., Akova Y.A., Benítez-Del-Castillo J., Boboridis K.G., Merayo-Lloves J., Rolando M., Labetoulle M. (2016). Revisiting the Vicious Circle of Dry Eye Disease: A Focus on the Pathophysiology of Meibomian Gland Dysfunction. Br. J. Ophthalmol..

[55] Tomlinson A., Bron A.J., Korb D.R., Amano S., Paugh J.R., Ian Pearce E., Yee R., Yokoi N., Arita R., Dogru M. (2011). The International Workshop on Meibomian Gland Dysfunction: Report of the Diagnosis Subcommittee. Investig. Ophthalmol. Vis. Sci..

[56] Holopainen J.M., Rantamäki A.H., Wiedmer S.K. (2013). Melting Points—The Key to the Anti-Evaporative Effect of the Tear Film Wax Esters. Investig. Ophthalmol. Vis. Sci..

[57] Tiffany J.M. (1994). Composition and Biophysical Properties of the Tear Film: Knowledge and Uncertainty. Adv. Exp. Med. Biol..

[58] Agarwal P., Craig J.P., Rupenthal I.D. (2021). Formulation Considerations for the Management of Dry Eye Disease. Pharmaceutics.

[59] Ewen King-Smith P., Hinel E.A., Nichols J.J. (2010). Application of a Novel Interferometric Method to Investigate the Relation between Lipid Layer Thickness and Tear Film Thinning. Investig. Ophthalmol. Vis. Sci..

[60] Viitaja T., Moilanen J., Svedström K.J., Ekholm F.S., Paananen R.O. (2021). Tear Film Lipid Layer Structure: Self-Assembly of O-Acyl-ω-Hydroxy Fatty Acids and Wax Esters into Evaporation-Resistant Monolayers. Nano Lett..

[61] Paananen R.O., Javanainen M., Holopainen J.M., Vattulainen I. (2019). Crystalline Wax Esters Regulate the Evaporation Resistance of Tear Film Lipid Layers Associated with Dry Eye Syndrome. J. Phys. Chem. Lett..

[62] Sane P., Salonen E., Falck E., Repakova J., Tuomisto F., Holopainen J.M., Vattulainen I. (2009). Probing Biomembranes with Positrons. J. Phys. Chem. B.

[63] Mouritsen O.G., Zuckermann M.J. (1985). Softening of Lipid Bilayers. Eur. Biophys. J..

[64] Kimball S.H., King-Smith P.E., Nichols J.J. (2010). Evidence for the Major Contribution of Evaporation to Tear Film Thinning between Blinks. Investig. Ophthalmol. Vis. Sci..

[65] Lemp M.A., Baudouin C., Baum J., Dogru M., Foulks G.N., Kinoshita S., Laibson P., McCulley J., Murube J., Pflugfelder S.C. (2007). The Definition and Classification of Dry Eye Disease: Report of the Definition and Classification Subcommittee of the International Dry Eye WorkShop (2007). Ocul. Surf..

[66] Potvin R., Makari S., Rapuano C.J. (2015). Tear Film Osmolarity and Dry Eye Disease: A Review of the Literature. Clin. Ophthalmol..

[67] Tsubota K. (1998). Tear Dynamics and Dry Eye. Prog. Retin. Eye Res..

[68] Chew C.K.S., Jansweijer C., Tiffany J.M., Dikstein S., Bron A.J. (1993). An Instrument for Quantifying Meibomian Lipid on the Lid Margin: The Meibometer. Curr. Eye Res..

[69] Cui K.W., Myung D.J., Fuller G.G. (2022). Tear Film Stability as a Function of Tunable Mucin Concentration Attached to Supported Lipid Bilayers. J. Phys. Chem. B.

[70] Wizert A., Iskander D.R., Cwiklik L. (2017). Interaction of Lysozyme with a Tear Film Lipid Layer Model: A Molecular Dynamics Simulation Study. Biochim. Biophys. Acta (BBA)-Biomembr..

[71] Georgiev G.A., Eftimov P., Yokoi N. (2019). Contribution of Mucins towards the Physical Properties of the Tear Film: A Modern Update. Int. J. Mol. Sci..

[72] Ring M.H., Rabensteiner D.F., Horwath-Winter J., Boldin I., Hörantner R., Haslwanter T. (2012). Introducing a New Parameter for the Assessment of the Tear Film Lipid Layer. Investig. Ophthalmol. Vis. Sci..

[73] Grasso A., Di Zazzo A., Giannaccare G., Sung J., Inomata T., Shih K.C., Micera A., Gaudenzi D., Spelta S., Romeo M.A. (2021). Sex Hormones Related Ocular Dryness in Breast Cancer Women. J. Clin. Med..

[74] Rolando M., Barabino S., Mingari C., Moretti S., Giuffrida S., Calabria G. (2005). Distribution of Conjunctival HLA-DR Expression and the Pathogenesis of Damage in Early Dry Eyes. Cornea.

[75] Aragona P., Giannaccare G., Mencucci R., Rubino P., Cantera E., Rolando M. (2021). Modern Approach to the Treatment of Dry Eye, a Complex Multifactorial Disease: A P.I.C.A.S.S.O. Board Review. Br. J. Ophthalmol..

[76] Uchino Y., Kawakita T., Miyazawa M., Ishii T., Onouchi H., Yasuda K., Ogawa Y., Shimmura S., Ishii N., Tsubota K. (2012). Oxidative Stress Induced Inflammation Initiates Functional Decline of Tear Production. PLoS ONE.

[77] Beranova L., Cwiklik L., Jurkiewicz P., Hof M., Jungwirth P. (2010). Oxidation Changes Physical Properties of Phospholipid Bilayers: Fluorescence Spectroscopy and Molecular Simulations. Langmuir.

[78] Wong-Ekkabut J., Xu Z., Triampo W., Tang I.M., Tieleman D.P., Monticelli L. (2007). Effect of Lipid Peroxidation on the Properties of Lipid Bilayers: A Molecular Dynamics Study. Biophys. J..

[79] Yusupov M., Van der Paal J., Neyts E.C., Bogaerts A. (2017). Synergistic Effect of Electric Field and Lipid Oxidation on the Permeability of Cell Membranes. Biochim. Biophys. Acta Gen. Subj..

[80] Wiczew D., Szulc N., Tarek M. (2021). Molecular dynamics simulations of the effects of lipid oxidation on the permeability of cell membranes. Bioelectrochemistry.

[81] Lis M., Wizert A., Przybylo M., Langner M., Swiatek J., Jungwirth P., Cwiklik L. (2011). The Effect of Lipid Oxidation on the Water Permeability of Phospholipids Bilayers. Phys. Chem. Chem. Phys..

[82] Bruch R.C., Thayer W.S. (1983). Differential Effect of Lipid Peroxidation on Membrane Fluidity as Determined by Electron Spin Resonance Probes. Biochim. Biophys. Acta.

[83] Yadav D.K., Kumar S., Choi E.H., Chaudhary S., Kim M.H. (2019). Molecular Dynamic Simulations of Oxidized Skin Lipid Bilayer and Permeability of Reactive Oxygen Species. Sci. Rep..

[84] Zhong J.Y., Lee Y.C., Hsieh C.J., Tseng C.C., Yiin L.M. (2018). Association between Dry Eye Disease, Air Pollution and Weather Changes in Taiwan. Int. J. Environ. Res. Public Health.

[85] Abusharha A.A., Pearce E.I., Fagehi R. (2016). Effect of Ambient Temperature on the Human Tear Film. Eye Contact Lens.

[86] Ho W.T., Chiu C.Y., Chang S.W. (2021). Low Ambient Temperature Correlates with the Severity of Dry Eye Symptoms. Taiwan J. Ophthalmol..

[87] Butovich I.A., Arciniega J.C., Wojtowicz J.C. (2010). Meibomian Lipid Films and the Impact of Temperature. Investig. Ophthalmol. Vis. Sci..

[88] Hirata H., Meng I.D. (2010). Cold-Sensitive Corneal Afferents Respond to a Variety of Ocular Stimuli Central to Tear Production: Implications for Dry Eye Disease. Investig. Ophthalmol. Vis. Sci..

[89] Wolkoff P. (2017). External Eye Symptoms in Indoor Environments. Indoor Air.

[90] Argüeso P. (2022). Human ocular mucins: The endowed guardians of sight. Adv. Drug Deliv. Rev..

[91] Rolando M., Iester M., Macrí A., Calabria G. (1998). Low Spatial-Contrast Sensitivity in Dry Eyes. Cornea.

[92] Cutrupi F., De Luca A., Di Zazzo A., Micera A., Coassin M., Bonini S. (2023). Real Life Impact of Dry Eye Disease. Semin. Ophthalmol..

[93] Ma J., Wei S., Jiang X., Chou Y., Wang Y., Hao R., Yang J., Li X. (2020). Evaluation of Objective Visual Quality in Dry Eye Disease and Corneal Nerve Changes. Int. Ophthalmol..

[94] Mitchell T., Murri M., Pflugfelder S.C. (2021). Video Viewing Blink Rate in Normal and Dry Eyes. Eye Contact Lens.

[95] Biousse V., Skibell B.C., Watts R.L., Loupe D.N., Drews-Botsch C., Newman N.J. (2004). Ophthalmologic Features of Parkinson’s Disease. Neurology.

[96] Di Zazzo A., Coassin M., Micera A., Mori T., De Piano M., Scartozzi L., Sgrulletta R., Bonini S. (2021). Ocular Surface Diabetic Disease: A Neurogenic Condition?. Ocul. Surf..

[97] Antonini M., Gaudenzi D., Spelta S., Sborgia G., Poddi M., Micera A., Sgrulletta R., Coassin M., Di Zazzo A. (2021). Ocular Surface Failure in Urban Syndrome. J. Clin. Med..

[98] Leonardi A., Lanier B. (2008). Urban eye allergy syndrome: A new clinical entity?. Curr. Med. Res. Opin..

[99] Sacchetti M., Lambiase A., Aronni S., Griggi T., Ribatti V., Bonini S., Bonini S. (2006). Hyperosmolar conjunctival provocation for the evaluation of nonspecific hyperreactivity in healthy patients and patients with allergy. J. Allergy Clin. Immunol..

[100] Gaudenzi D., Mori T., Crugliano S., Grasso A., Frontini C., Carducci A., Yadav S., Sgrulletta R., Schena E., Coassin M. (2022). AS-OCT and Ocular Hygrometer as Innovative Tools in Dry Eye Disease Diagnosis. Appl. Sci..

[101] De Luca A., Ferraro A., De Gregorio C., Laborante M., Coassin M., Sgrulletta R., Di Zazzo A. (2023). Promising High-Tech Devices in Dry Eye Disease Diagnosis. Life.

[102] Rolando M., Cantera E., Mencucci R., Rubino P., Aragona P. (2018). The Correct Diagnosis and Therapeutic Management of Tear Dysfunction: Recommendations of the P.I.C.A.S.S.O. Board. Int. Ophthalmol..

[103] Wolffsohn J.S., Arita R., Chalmers R., Djalilian A., Dogru M., Dumbleton K., Gupta P.K., Karpecki P., Lazreg S., Pult H. (2017). TFOS DEWS II Diagnostic Methodology Report. Ocul. Surf..

[104] Gilbard J.P., Farris R.L., Santamaria J. (1978). Osmolarity of Tear Microvolumes in Keratoconjunctivitis Sicca. Arch. Ophthalmol..

[105] McDonald J.E., Brubaker S. (1971). Meniscus-Induced Thinning of Tear Films. Am. J. Ophthalmol..

[106] Berger R.E., Corrsin S. (1974). A Surface Tension Gradient Mechanism for Driving the Pre-Corneal Tear Film after a Blink. J. Biomech..

[107] Bron A.J., Tiffany J.M., Yokoi N., Gouveia S.M. (2002). Using Osmolarity to Diagnose Dry Eye: A Compartmental Hypothesis and Review of Our Assumptions. Adv. Exp. Med. Biol..

[108] Liu D.T.S., Di Pascuale M.A., Sawai J., Gao Y.Y., Tseng S.C.G. (2005). Tear Film Dynamics in Floppy Eyelid Syndrome. Investig. Ophthalmol. Vis. Sci..

[109] McCulley J.P., Sciallis G.F. (1977). Meibomian Keratoconjunctivitis. Am. J. Ophthalmol..

[110] Mcmonnies C.W. (2018). Tear instability importance, mechanisms, validity and reliability of assessment. J. Optom..

[111] Endo K., Goto E., Suzuki A., Fujikura Y., Tsubota K. (2002). Innovative Dry Eye Diagnosis System Using Microbalance Technology. Adv. Exp. Med. Biol..

[112] Tomlinson A., Doane M.G., McFadyen A. (2009). Inputs and Outputs of the Lacrimal System: Review of Production and Evaporative Loss. Ocul. Surf..

[113] Vera J., Redondo B., Molina R., Jiménez R. (2022). Effects of Wearing Swimming Goggles on Non-Invasive Tear Break-up Time in a Laboratory Setting. J. Optom..

[114] Imhof R.E., De Jesus M.E.P., Xiao P., Ciortea L.I., Berg E.P. (2009). Closed-Chamber Transepidermal Water Loss Measurement: Microclimate, Calibration and Performance. Int. J. Cosmet. Sci..

[115] Purslow C., Wolffsohn J.S. (2005). Ocular Surface Temperature: A Review. Eye Contact Lens.

[116] Mengher L.S., Bron A.J., Tonge S.R., Gilbert D.J. (1985). A Non-Invasive Instrument for Clinical Assessment of the Pre-Corneal Tear Film Stability. Curr. Eye Res..

[117] King-Smith P.E., Nichols J.J., Nichols K.K., Fink B.A., Braun R.J. (2008). Contributions of Evaporation and Other Mechanisms to Tear Film Thinning and Break-Up. Optom. Vis. Sci..

[118] King-Smith P.E., Fink B.A., Nichols J.J., Nichols K.K., Braun R.J., McFadden G.B. (2009). The Contribution of Lipid Layer Movement to Tear Film Thinning and Breakup. Investig. Ophthalmol. Vis. Sci..

[119] Korb D., Herman J. (1979). Corneal Staining Subsequent to Sequential Fluorescein Instillations. J. Am. Optom. Assoc..

[120] Goto E., Tseng S.C.G. (2003). Kinetic Analysis of Tear Interference Images in Aqueous Tear Deficiency Dry Eye before and after Punctal Occlusion. Investig. Ophthalmol. Vis. Sci..

[121] Arita R., Morishige N., Fujii T., Fukuoka S., Chung J.L., Seo K.Y., Itoh K. (2016). Tear Interferometric Patterns Reflect Clinical Tear Dynamics in Dry Eye Patients. Investig. Ophthalmol. Vis. Sci..

[122] Arita R. (2018). Meibography: A Japanese Perspective. Investig. Ophthalmol. Vis. Sci..

[123] Rolando M., Valente C., Barabino S. (2008). New Test to Quantify Lipid Layer Behavior in Healthy Subjects and Patients with Keratoconjunctivitis Sicca. Cornea.

[124] Korb D.R., Blackie C.A. (2008). Meibomian Gland Diagnostic Expressibility: Correlation with Dry Eye Symptoms and Gland Location. Cornea.

[125] Di Cello L., Pellegrini M., Vagge A., Borselli M., Desideri L.F., Scorcia V., Traverso C.E., Giannaccare G. (2021). Advances in the Noninvasive Diagnosis of Dry Eye Disease. Appl. Sci..

[126] Norm M. (1979). Semiquantitative Interference Study of Fatty Layer of Precorneal Film. Acta Ophthalmol..

[127] Hamano H., Hori M., Kawabe M. (1980). Clinical Applications of Biodifferential Interference Microscope. Contact Intraocular Lens Med. J..

[128] Hamano H., Hori M., Kawabe M. (1979). Biodifferential Interference Microscopic Observations on Anterior Segment of Eye. J. Jpn. Contact Lens Soc..

[129] Kilp H., Schmidt E., Kirchner L., Zipf-Pohl A., Holly F. (1986). Tear Film Observation by Reflecting Microscopy and Differential Interference Contrast Microscopy. The Preocular Tear Film in Health, Disease, and Contact Lens Wear.

[130] Josephson J. (1983). Appearance of the Preocular Tear Film Lipid Layer. Am. J. Optom. Physiol. Opt..

[131] Guillon J.P. (1982). Tear Film Photography and Contact Lens Wear. J. Br. Contact Lens Assoc..

[132] Doane M.G., Lee M.E. (1998). Tear Film Interferometry as a Diagnostic Tool for Evaluating Normal and Dry-Eye Tear Film. Adv. Exp. Med. Biol..

[133] Guillon M., Styles E., Guillon J.P., Cécile Maïssa M. (1997). Preocular Tear Film Characteristics of Nonwearers and Soft Contact Lens Wearers. Optom. Vis. Sci..

[134] Paulsen F.P., Schaudig U., Thale A.B. (2003). Drainage of Tears: Impact on the Ocular Surface and Lacrimal System. Ocul. Surf..

[135] Drouault-Holowacz S., Bieuvelet S., Burckel A., Rigal D., Dubray C., Lichon J.L., Bringer P., Pilon F., Chiambaretta F. (2009). Antioxidants Intake and Dry Eye Syndrome: A Crossover, Placebo-Controlled, Randomized Trial. Eur. J. Ophthalmol..

[136] Doan S., Chiambaretta F., Baudouin C. (2014). Evaluation of an Eyelid Warming Device (Blephasteam) for the Management of Ocular Surface Diseases in France: The ESPOIR Study. J. Français Ophtalmol..

[137] Lane S.S., Dubiner H.B., Epstein R.J., Ernest P.H., Greiner J.V., Hardten D.R., Holland E.J., Lemp M.A., Mcdonald Ii J.E., Silbert D.I. (2012). A New System, the LipiFlow, for the Treatment of Meibomian Gland Dysfunction (MGD). Cornea.

[138] Lee S.Y., Tong L. (2012). Lipid-Containing Lubricants for Dry Eye: A Systematic Review. Optom. Vis. Sci..

[139] Aparicio-Soto M., Sánchez-Hidalgo M., Rosillo M.Á., Castejón M.L., Alarcón-De-La-Lastra C. (2016). Extra Virgin Olive Oil: A Key Functional Food for Prevention of Immune-Inflammatory Diseases. Food Funct..

[140] George E.S., Marshall S., Mayr H.L., Trakman G.L., Tatucu-Babet O.A., Lassemillante A.C.M., Bramley A., Reddy A.J., Forsyth A., Tierney A.C. (2019). The Effect of High-Polyphenol Extra Virgin Olive Oil on Cardiovascular Risk Factors: A Systematic Review and Meta-Analysis. Crit. Rev. Food Sci. Nutr..

[141] Giannaccare G., Pellegrini M., Sebastiani S., Bernabei F., Roda M., Taroni L., Versura P., Campos E.C. (2019). Efficacy of Omega-3 Fatty Acid Supplementation for Treatment of Dry Eye Disease: A Meta-Analysis of Randomized Clinical Trials. Cornea.

[142] Giannaccare G., Taroni L., Senni C., Scorcia V. (2019). Intense Pulsed Light Therapy In The Treatment Of Meibomian Gland Dysfunction: Current Perspectives. Clin. Optom..

[143] Cote S., Zhang A.C., Ahmadzai V., Maleken A., Li C., Oppedisano J., Nair K., Busija L., Downie L.E. (2020). Intense Pulsed Light (IPL) Therapy for the Treatment of Meibomian Gland Dysfunction. Cochrane Database Syst. Rev..

[144] Magno M., Moschowits E., Arita R., Vehof J., Utheim T.P. (2021). Intraductal Meibomian Gland Probing and Its Efficacy in the Treatment of Meibomian Gland Dysfunction. Surv. Ophthalmol..

[145] Blackie C.A., Korb D.R. (2015). A Novel Lid Seal Evaluation: The Korb-Blackie Light Test. Eye Contact Lens.

